# Using Failure Mode, Effect and Criticality Analysis to Improve Safety in the COVID Mass Vaccination Campaign

**DOI:** 10.3390/vaccines9080866

**Published:** 2021-08-05

**Authors:** Alessandra Buja, Mariagiovanna Manfredi, Giuseppe De Luca, Chiara Zampieri, Sofia Zanovello, Davor Perkovic, Francesca Scotton, Anil Minnicelli, Anna De Polo, Vittorio Cristofori, Laura Biasi, Tatjana Baldovin, Chiara Bertoncello, Silvia Cocchio, Vincenzo Baldo

**Affiliations:** Department of Cardiological, Thoracic, Vascular Sciences and Public Health, University of Padua IT, 35131 Padua, Italy; alessandra.buja@unipd.it (A.B.); mariagiovanna.manfredi@studenti.unipd.it (M.M.); giuseppe.deluca.2@studenti.unipd.it (G.D.L.); sofia.zanovello@studenti.unipd.it (S.Z.); davor.perkovic.1@studenti.unipd.it (D.P.); francesca.scotton.1@studenti.unipd.it (F.S.); anil.minnicelli@studenti.unipd.it (A.M.); anna.depolo@studenti.unipd.it (A.D.P.); vittorio.cristofori@studenti.unipd.it (V.C.); laura.biasi@studenti.unipd.it (L.B.); tatjana.baldovin@unipd.it (T.B.); chiara.bertoncello@unipd.it (C.B.); silvia.cocchio@unipd.it (S.C.); vincenzo.baldo@unipd.it (V.B.)

**Keywords:** patient safety, proactive management, vaccination campaign, SARS-COV-2

## Abstract

Vaccination against SARS-CoV-2 will likely be the most promising way to combat the pandemic. Even if mass vaccination is urgent, it should still always be supported by appropriate patient safety management. The aim of this study, based on failure mode, effects and criticality analysis (FMECA), was to identify possible failures and highlight measures that can be adopted to prevent their occurrence. A team of resident doctors in public health from the University of Padua and specialists in risk analysis in public health examined the mass vaccination process. A diagram was drafted to illustrate the various phases of mass vaccination, analyze the process, and identify all failure modes. Criticalities were ascertained by rating the severity, frequency and likelihood of failure detection on a scale of 1 to 10. We identified a total of 71 possible faults distributed over the various phases of the process, and 34 of them were classified as carrying a high risk. For the potentially high-risk failure modes, we identified 63 recommended actions to contain the cause of their occurrence or improve their detection. For the purpose of detecting potential failures, FMECA can be successfully applied to mass vaccination, which should be considered a high-risk process.

## 1. Introduction

Deadly pandemics and large-scale epidemics have challenged human existence throughout history. While these crises were once separated by centuries, or at least many decades, they are now becoming much more common. Since 2003, we have experienced severe acute respiratory syndrome (SARS) (a near pandemic), an influenza pandemic (H1N1pdm in 2009), a chikungunya pandemic (2014), a Zika pandemic (2015), and a widespread pandemic-like extension of Ebola over five African countries, with cases exported globally (2014 to 2015) [1].

SARS-CoV-2 is a new type of coronavirus that causes a serious, contagious disease (COVID-19) [2]. It was first reported in Wuhan, China in November 2019 and WHO confirmed the first case on 31 December 2019. Since then, it has been spreading rapidly and the outbreak was declared a global pandemic on 11 March 2020 [3,4]. The rapid worldwide spread of the virus has resulted in over 140 million people becoming infected and more than 3 million deaths (data updated to April 2021), causing a global health, social and economic crisis [5].

Although hundreds of clinical trials have been initiated since the outbreak of COVID-19, an antiviral drug that is effective in all patient groups has yet to be developed and assessed. Hence the urgent need to vaccinate the whole population against the SARS-CoV-2 virus. Vaccination will likely be the most effective way to counter the COVID-19 pandemic [6]. The process of drug discovery is costly and time-consuming. Only widespread preventative vaccination can help to lower the burden of the pandemic, playing a pivotal part in efficiently and sustainably protecting people from viral infections, and either eliminating or significantly reducing their transmission within the population [7]. Inducing herd immunity by means of mass vaccination programs has been a very successful strategy for preventing the spread of other infectious diseases. It also protects the most vulnerable population groups unable to develop immunity, such as individuals with immunodeficiencies or a weakened immune system due to underlying medical or debilitating conditions [8].

While the development of a safe and effective COVID-19 vaccine has not been easy, its production and distribution, and its administration especially, also pose extraordinary challenges [6]. Mass vaccination can be seen as a critical process because of the complexity of the organizational machine needed to manage huge numbers of people as quickly as possible, in other spaces as well as healthcare facilities, while always maintaining patient safety standards.

Two methods are used to manage the risks associated with critical processes. One is reactive: measures are taken after an adverse event has occurred to prevent it from happening again. The other is proactive: processes are analyzed a priori to prevent adverse events from occurring in the first place. One of the most popular proactive methods is failure mode, effect and criticality analysis (FMECA). According to the Joint Commission, FMECA is a systematic, analytical technique used prospectively by a team to prevent the appearance of problems associated with a process before they occur [9]. This is a step-by-step approach to identifying “failures” that lead to poor-quality, unsafe, unreliable, or inefficient care [10]. It is based on the concept that a risk is related not only to the likelihood of a failure occurring, but also to the severity of the failure’s consequences and the feasibility of detecting and intercepting a failure before it occurs. The FMECA approach provides a systematic way to identify failures, and also prioritizes the most important ones for improvement [11]. It was originally developed by engineers to study complex systems and is typically applied to high-risk industries, such as nuclear power generation and commercial aviation, where an error can have very serious consequences [12,13].

These step-by-step assessment methods have increasingly been used to maximize the safety and quality of clinical care in many healthcare settings and are now widely used to proactively evaluate complex clinical processes.

The purpose of this study was to conduct a prospective and systematic analysis of the various stages of the mass vaccination process, applying FMECA to identify possible errors and enable preemptive measures to be taken.

## 2. Materials and Methods

**Figure 1:**
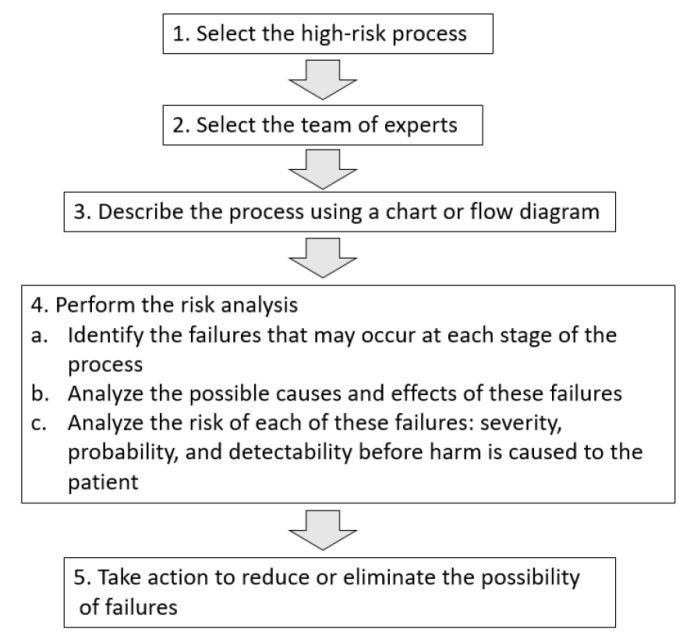
Steps involved in FMECA.

The FMECA was conducted in accordance with the process described in Figure 1.

The analysis was conducted in April 2021, about 4 months after the start of the COVID-19 vaccination campaign in Italy when about 7,401,431 people (12.3% of the total population) had received a single dose of vaccine while 3,333,644 (5.5% of the total population) had completed the vaccination course [14].

**Figure 2:**
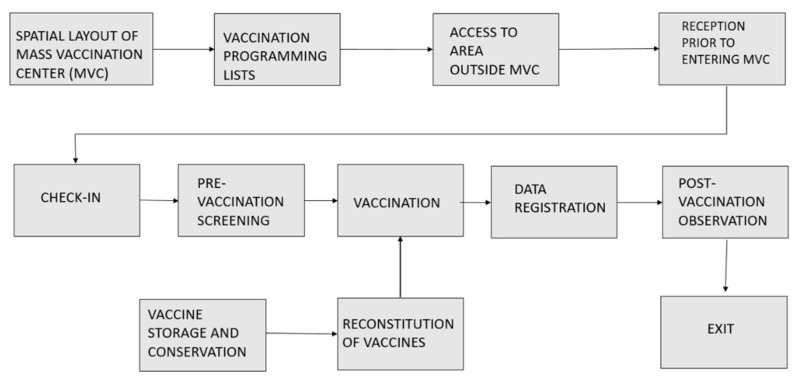
Phases of the mass vaccination process.

After an introductory session held with a team of experts to explain the features of FMECA, mass vaccination against SARS-CoV-2 was identified as the high-risk process to investigate. The team consisted of resident doctors in public health from the University of Padua who were actively involved in the mass vaccination program underway in Veneto (Italy) in different local health units, together with in public health specialists with expertise in risk analysis. A process diagram was drawn up, illustrating the various phases of the mass vaccination process (Figure 2).

Analyzing the process enabled all failure modes to be identified, and criticalities were pinpointed by rating these failure modes in terms of their: occurrence (probability of the event occurring); detectability (probability of the event going undetected, and of it reaching the patient); and severity (effect of the error on the patient). Each item was classified on a scale of 1 to 10. Table 1 shows the rating scale used in this study, which is an adapted version of those used in already-published studies. In particular, to quantify the potential effects of a failure mode associated with the loss of vaccine and delays in the vaccination of prioritized population groups, a rating higher than 7 was assigned to the severity of all the related failure modes. The same applied to all events that were not an immediate source of danger to users, but were potential sources of contagion, such as gatherings of people failing to comply with social distancing requirements. For each possible failure, five team members assigned a score to the three rating scales for severity, frequency and likelihood of detection.

The numerical score quantifying the three items was used to calculate a risk priority number (RPN), which is the product of the severity, occurrence and detectability scores (RPN = occurrence × detectability × severity). The median value of the failures’ RPNs was used to assess the final score, and we also reported the ranges of the risk priority numbers (RPN).

Thus, as part of the FMECA, the RPN is a numerical estimate of a risk attributed to a process, or a step in a process; it identifies the elements most likely to contribute to medically serious failures.

The maximum RPN is 1000 and any RPN > 100 was used to identify a high-risk failure [15]. This is a conventional value that is only used to prioritize failures, but all failures should be addressed, albeit more or less promptly.

For each failure mode, the team analyzed the criticality index and decided whether the risk was acceptable or improvements were needed. The highest scoring failures were considered high-risk and identified as priority areas in which improvements, through safety strategies, should be made.

## 3. Results

The assessment process detected a total of 71 possible failures distributed over the various phases of the process (Figure 2), and 34 of them were classified as high-risk. The phases with the largest number of possible failures were: “spatial layout of vaccination center (MVC)” (14 failures); and “pre-vaccination screening” (13 failures).

The RPNs obtained (Table 2) ranged from 10 to 378. Every phase of the mass vaccination process, with the exception of “access to area outside vaccination center”, “reception prior to entering vaccination center” and “exit”, were potentially critical with RPNs > 100. The most important failures producing the highest RPNs were found in the “post-vaccination observation” and “pre-vaccination screening” phases. They included, for instance, “Failure to investigate the clinical history (e.g., previous positivization, prior anaphylaxis for vaccine components)”, “Erroneous medical details provided by user” and “Users leave MVC before completing the required observation period (based on their risk factors)”.

For the potential high-risk failure modes, at least one recommended action was identified with a view to reducing their occurrence or improving their detection. This resulted in the 63 recommendations listed in Table 3.

## 4. Discussion

FMECA is increasingly used as a method for assessing processes and improving their safety. Given the numerous potential failure modes in every phase of a mass vaccination process (most of them defined as high-risk), our study shows that this activity should be considered a high-risk process, just like other healthcare activities conducted in ambulatory settings.

Many failures are related to the spatial layout of the MVC. The unavailability of health facilities with the features needed to conduct mass vaccination programs, and the unfeasibility of quickly building new ones made it objectively difficult to reconcile the need to ensure user safety with the adaptation of spaces normally used for other activities.

Taking into account the operational document for organizing MVCs published in the Veneto Regional Authority’s official bulletin (Bur) in March 2021 [16] and the latest interim guidance published by the World Health Organization (WHO) [17], our aim was to draw up a list of actions considering all the architectural variables (flooring, lighting, alternative routes for the disabled, solutions to guarantee social distancing) involved in adapting spaces normally used for other activities but able to accommodate large numbers of people and ensure patient safety.

In the phase where the vaccines are reconstituted, the RPNs were ≥200 for every failure mode identified (“Incorrect identification of vials”, “Errors in dilution procedure”, “Failure to recognize vials of vaccine to be rejected”, and “Failure to identify use-by date and time”). This finding could be due to the complexity of the procedures and the high workflow typical of mass vaccination resulting in failures that are hard to detect but have potentially severe effects on public health (by reducing the efficacy of vaccination) and users’ health (by causing anaphylactic reactions). For many years, the emphasis has been on the adverse effects of vaccines after their administration, while little is known about the delicate phase of vaccine dose preparation. Paparella’s work [18] highlights the importance of the various errors occurring in this phase of vaccination programs. Our work replaces her findings because one of the most commonly reported errors concerned the failure to identify the use-by date and time on a vial of vaccine. Strategies to avoid confusion and harm to users have already been published, such as: weekly checks for expired vaccines as well as each time a vial is collected from the stock; avoiding waste by rotating stock to ensure that the first in is the first out; checking people’s age and comorbidities before selecting and administering vaccines; and keeping track of immunization schedules. Considering their significant impact and the different settings and workflows analyzed, we propose some additional prevention strategies, such as: dedicating a room that is physically separate from the clinical area; using separate workstations with dedicated staff for the various types of vaccine; creating operating instructions and having flowcharts on display; writing the date and time of thawing on the vial; checking that all vials have labels showing the use-by date and time; and training staff on the reconstitution of the different types of vaccine.

Judging from the RPNs, the pre-vaccination screening and post-vaccination observation phases should be considered the phases at highest risk: “Failure to investigate clinical history (e.g., prior anaphylaxis for vaccine components)” or “Users leave MVC before completing the required observation period (based on their risk factors)” could have the most severe effects on users (adverse events in an unprotected environment, or a patient’s death). We recommend several actions to prevent these most risky failures, such as giving users written notification of their exit time, checking users at the exit, or giving users precise indications on potential symptoms of an adverse reaction. The most important recommendation, however (also contained in the COVID-19 Vaccination Program: Guidance for Healthcare Practitioners published by Public Health England [19]), is to ensure that staff are continuously given updated training on COVID-19 vaccine contraindications and precautions.

As the RPNs show, the vaccination phase also deserves healthcare workers’ utmost attention. A systematic review of the medical literature showed that the most common vaccination-related error involved the administration of the wrong vaccine. Several trends were identified relating to this issue, including the incorrect administration of vaccine products with similar names or products licensed for use in specific age groups. Vaccines with different age-based formulations tended to be more often associated with administration errors than vaccines with a single dosage for all ages. Although most errors were reported as having caused “no adverse reaction” [20], we judged that the same failure should be considered a high-risk failure because of the presence of specific recommendation for different age and comorbidity groups. We recommend some actions to take, including “Organize separate lines for different vaccines, with clearly-marked routes”, and “Accurately check the vaccine prescription and register the type of vaccine administered (equip outpatient clinics with IT support)”.

To the best of our knowledge, this study is the first to have applied FMECA to the mass vaccination process and the organization of an MVC with a view to making recommendations for improvements to ensure the safety of the mass vaccination process.

FMECA is liable to several limitations, the main one being the unavoidably subjective selection of the failure modes and calculation of the criticality indexes. To minimize this problem, explicit criteria were stipulated for assessing the frequency, severity and detectability of failures, and every failure was discussed by all of the team members. That said, although the failures reported could have external validity and be more broadly applicable elsewhere, the heterogeneity of healthcare systems and structural features in different countries (regarding the availability of information technology systems, for instance) makes the definition of RPN-based scores context-specific (e.g., healthcare systems supported by technology but without integrated and unified databases). In further research on the topic of FMECA, we suggest interviewing personnel employed in other countries in order to draw up a more generalized model.

## 5. Conclusions

FMECA can be usefully performed on a mass vaccination process to help identify potential failures. Safety strategies were recommended for each failure mode identified by our analysis and these recommended actions could be considered for practice and for further studies in the field.

## Figures and Tables

**Table 1:** Rating scales for severity, frequency and likelihood of detection.

Severity of the Effect of the Failure Mode	Rating
Slight annoyance: may affect the system	1
Moderate system problem: may affect the patient	2–3
Major system problem: may affect the patient	4–5
Minor injury	6–7
Major injury	8
Terminal injury or death	9–10
Failure mode frequency in the vaccination center	Rating
Once a year	1
Once a month	2–4
Once a week	5–6
Once a day	7–8
Several times a day	9–10
Likelihood of detecting a failure mode when it occurs	Rating
≥90%	1
80%	2
70%	3
60%	4
50%	5
40%	6
30%	7
20%	8
10%	9
0%	10

**Table 2:** Possible failures at each stage of the process and corresponding RPNs, ranked by RPN in each stage.

Stage and Possible Failure	RPN (Medians and Ranges)
**Spatial Layout of Mass Vaccination Center (MVC)**	
Inadequate emergency equipment	216 (200–350)
Inadequate flooring and stairs (risk of falls)	70 (24–240)
Architectural barriers	60 (35–84)
Inadequate fire protection	54 (30–100)
Unavailability of hand sanitizer, properly-equipped handwashing stations at entrance to MVC and health facilities, or personal protective equipment	42 (24–225)
Inadequate microclimatic comfort	42 (10–60)
Inadequate route signage-emergency services (118)	40 (20–42)
Inadequate disaster recovery plan	30 (25–100)
Inadequate spaces reserved for toilets	30 (8–50)
Inadequate web security	24 (5–40)
Inadequate lighting	16 (12–48)
Inadequate space for personnel refreshment	16 (10–25)
Inadequate web connection	16 (4–28)
Inadequate electronic security and uninterruptible power supply	15 (4–32)
**Vaccination Programming Lists**	
Missed appointments for second doses	192 (96–200)
Failure to call users included in target categories	168 (80–360)
Wrong timing for scheduling second doses (second doses given too soon or too late vis-à-vis the recommended interval between doses)	140 (96–280)
Incorrect user prioritization	60 (54–140)
Inadequate remainder and recall (vaccination appointments and defaulting)	42 (9–60)
Overbooking	18 (16–54)
Invitations sent out to users already vaccinated	18 (9–42)
**Access to Area Outside MVC**	
Insufficient parking space	27 (10–54)
Inadequate signage to follow at the MVC	20 (10–105)
**Reception Prior to Entering MVC**	
Failure to check users’ temperature at the gate	56 (7–120)
Inadequate waiting space for repeating temperature check in event of high temperature on first measurement	42 (20–84)
Unavailability of refreshments for users or protection against the weather for waiting users	30 (12–90)
Gatherings	18 (10–48)
Presence of unnecessary companions	10 (7–60)
**Check-In (personal identification and documentation of appointment)**	
Failure to identify users (rejection of users)	240 (72–245)
Admission of people not eligible for vaccination	72 (64–112)
Insufficient waiting space for check-in	60 (42–96)
**Vaccine Storage and Conservation**	
Incorrect defrosting of vaccine vials	160 (98–200)
Ineffective monitoring of vaccine vial expiry dates	150 (84–160)
Inadequate vaccine preparation for supply or distribution	128 (28–240)
Ineffective temperature control in storage	105 (56–160)
Ineffective stock/inventory management	63 (48–112)
Underestimation of refrigerators needed to store the various types of vaccine	60 (20–160)
Inadequate anti-theft protection	45 (14–160)
**Reconstitution of Vaccines**	
Incorrect identification of vials (in event of simultaneous presence of different types of vaccine at the same vaccination session)	288 (224–384)
Errors in dilution procedure	216 (80–240)
Failure to recognize vials of vaccine to be rejected (defective, discolored, particulate matter, etc.)	200 (80–288)
Failure to identify use-by date and time	200 (80–288)
**Pre-Vaccination Screening**	
Failure to investigate clinical history (e.g., previous positivization, prior anaphylaxis for vaccine components)	378 (80–512)
Erroneous medical details provided by user	360 (216–512)
Failure to assign correct type of vaccination based on contraindications derived from medical history	324 (144–384)
Users’ failure to provide vaccination history (e.g., previous vaccination, type of vaccination, previous adverse effects)	300 (240–343)
Failure to detect contraindications to vaccine (early pregnancy)	240 (120–280)
Incomplete medical history collection	216 (128–360)
Failure to notify operators assigned to post-vaccination observation of any clinical conditions that require an extension of the post-vaccination observation period	200 (180–343)
Failure to collect users’ completed consent forms	108 (90–144)
Users’ failure to understand due to unreliable information provided by healthcare personnel, or to language or comprehension issues	100 (80–384)
Failure to advise vaccine recipients about possible adverse reactions and how to report them, and time to develop immunity	80 (64–210)
Insufficient space and equipment for medical history to be collected	36 (30–54)
Insufficient space to wait for medical history to be collected	36 (30–48)
Gatherings in waiting rooms	18 (16–112)
**Vaccination**	
Administration of the wrong type of vaccine (different COVID-19 vaccines given for the first and second doses, wrong type of vaccine based on user’s age, comorbidities, prescribed medication)	280 (192–336)
Inadvertent administration of the whole multi-dose vial of vaccine instead of the recommended dose	224 (98–336)
Administration of an incomplete dose of vaccine (quantity)	168 (112–216)
Inadvertent administration of over-diluted vaccine	140 (112–168)
Inadvertent administration of the diluent alone (for COVID-19 vaccines requiring dilution)	128 (56–240)
**Data Registration**	
Wrong scheduling of second dose	160 (120–288)
Wrong type of vaccine registered	144 (60–288)
Wrong person registered as having been vaccinated	144 (60–180)
Delayed registration (by days or weeks)	120 (64–288)
Failure to register vaccination	120 (32–150)
Failure to deliver vaccination certificate	32 (30–126)
**Post-Vaccination Observation**	
Users leave MVC before completing the required observation period (based on their risk factors)	360 (288–480)
Inadequate management of adverse reactions during observation period	140 (72–300)
Inadequate waiting space for post-vaccination observation	64 (45–90)
**Exit**	
Inadequate outflow of pedestrian users from the MVC	42 (32–50)

**Table 3:** Actions recommended to improve the mass vaccination system.

Process Step	Recommended Actions
Spatial layout of MVC	Preparing a disaster recovery plan Signaling areas with inadequate flooring, notifying the technical office for repairs, identifying alternative routes Identifying alternative routes for users with disabilities Improving signage and removing architectural barriers Ensuring availability of hand sanitizer or equipped handwashing stations at entrance to vaccination sites and health facilities Installing and monitoring air conditioning system Periodically checking web security and connection Installing temporary lighting fixtures to ensure adequate local lighting Providing a backup alternative current generator
Vaccination programming lists	Reminders with correct dates of appointments for vaccination Opportunity for self-booking by users belonging to previously-validated lists (selection based personal data or prescription charge exemptions, or lists of workers) Creating automated date calculators and dedicated workstations to notify users of appointments for second doses of vaccine before they leave Cross-referencing of users’ tax codes with prescription charge exemption database for diseases and pharmaceutical flows Cross-referencing of users’ tax codes with their vaccination history Cross-referencing of users’ tax codes with results of SARS-CoV-2 tests (to identify previous COVID patients, who require only one dose of vaccine)
Access to area outside MVC	Suitable choice of location for MVC, taking parking space into account Adequate signage of user pathways
Reception prior to entering MVC	Organize periodic check on instrumental equipment for triage access Identify separate outside space for users with high body temperature (BT) awaiting second temperature check Adequately marking routes with markers for distancing, and for directing users where to go
Check-in (personal identification and documentation of appointment)	Create clear and standardized communication channels for the whole team conducting the vaccination campaign. Avoid using oral communications or unofficial channels (e.g., use mail, not messages) Diversify tasks for colleagues during a single shift to improve concentration Avoid permissiveness in reception phase (e.g., clear criteria regarding accompanying persons eligible for vaccination)
Vaccine storage and conservation	Staff training on general storage and handling principles, and standard operating procedures for vaccine management Designate a primary vaccine coordinator, responsible for ensuring all vaccines are stored and handled correctly Adequacy of refrigerator maintenance program Provide for remote temperature control Ensure the manager/pharmacist to call in the event of an alarm can be reached by phone Training of staff loading-unloading vaccines Automated report submission Monitoring of vaccine transport contract based on a checklist Automatic reporting on daily stocks to the MVC manager Equipment check list for warehouse handling vehicles and containers Alarms connected with a security service
Reconstitution of vaccines	Storage in separate refrigerators with clearly-visible labels/signs: use different colors, and avoid waste by rotating stock to ensure the first in is the first out Organize vaccination sessions with no more than 2 types of vaccine Staff training on reconstitution of different types of vaccine Suitable environment: minimize distractions; use a dedicated room, physically separated from the clinical activities; use separate workstations with dedicated staff for the various types of vaccine; prepare all the materials needed to dilute 1 bottle with a final check (e.g., that 6 syringes have actually been filled from 1 Pfizer vial) Create operating instructions and have flowcharts on display Write the date and time of thawing on the vial and check that all vials have been labeled with the “use-by” date and time
Pre-vaccination screening	Adequate choice of space for collecting users’ medical histories to guarantee privacy and quiet Advise users to bring a signed consent form and medical history questionnaire, possibly completed with the help of their GP Include a remainder to bring personal clinical records of chronic conditions in messages about vaccination appointments Continuously update staff training on contraindications to COVID-19 vaccine (e.g., systemic allergic reactions to a previous dose of the same vaccine and/or any components/excipients; episodes of heparin-induced thrombocytopenia and thrombosis (HITT or HIT type 2); a clotting episode with concomitant thrombocytopenia following a first dose of AstraZeneca vaccine and precautions (e.g., current or previous COVID-19 disease, long COVID-19 symptoms, interval between treatment for COVID-19 and vaccination, pregnancy, breastfeeding). Minor illnesses without fever or systemic symptoms are not valid reasons to postpone immunization. Staff training on the choice of the type of vaccine to administer, considering age, comorbidities (e.g., bleeding disorders, cancer, immunosuppression) and drugs (e.g., anticoagulants) Use checklist to collect medical history complete with information on the COVID area Check type and time of previous vaccinations (if administering a second dose) Materials such as brochures and resource kits can help in communicating with users or caregivers regarding the benefits and risks of vaccination, and behaviors required
Vaccination	Accurately check the vaccine prescription and register the type of vaccine administered (equip outpatient clinics with IT support) Organize separate lines for different vaccines, with clearly-marked routes Staff training on injection technique (area for injection, checking quantity of vaccine administered, type of needle)
Data registration	Position the registration area in a suitable place (away from noisy areas) Rotate personnel in charge of data recording Training and updating program for personnel in charge of recording vaccination data; registration in the presence of users during the observation period Checklist for ensuring the maintenance and availability of IT support
Post-vaccination observation	Giving users written notification of their exit time Checking users at the exit Informing users of the first symptoms for which they should alert the staff Ensuring emergency trolleys are easily accessible and fully equipped Staff training on period of observation after vaccination and on basic life support and defibrillation Presence of a first aid point at the MVC Social distancing in waiting spaces and rest area
Exit	Adequate signage to ensure the regular flow of people towards the exit of the MVC

## Data Availability

The datasets analyzed during the current study are not publicly available but are available from the corresponding author on reasonable request.
